# Uremia-induced lysine modifications transform plasma albumin into a high-density lipoprotein receptor inhibitor

**DOI:** 10.1186/2050-6511-13-S1-A40

**Published:** 2012-09-17

**Authors:** Veronika Binder, Michael Holzer, Dalia El-Gamal, Sabine Dirnberger, Gunther Marsche

**Affiliations:** 1 Institute of Experimental and Clinical Pharmacology, Medical University of Graz, 8010 Graz, Austria

## Background

Protein damage induced by retained uremic solutes may be an important component in the pathophysiology of advanced renal disease. Albumin isolated from hemodialysis patients was recently shown to block high-density lipoprotein (HDL) receptor-mediated cholesterol uptake. However, post-translational modifications that render albumin a scavenger receptor class B type I (SR-BI) ligand are not known. We hypothesized that the elimination of positive charge through oxidation of albumin-lysine residues is required to generate recognition motifs for SR-BI. Since carbamylation and carboxymethylation are major lysine modifications *in vivo*, we aimed at investigating their influence on the binding properties of HD-albumin to SR-BI.

## Methods

Albumin from HD patients and control subjects was isolated from serum by affinity chromatography. Mass spectrometry was used to study structurally defined lysine modifications on HD-albumin. Competition experiments (displacement of Alexa-labeled HDL) were performed to assess binding affinity of modified albumin to SR-BI.

## Results

We identified a significant increase in 3-chlorotyrosine, carbamyllysine and carboxymethyllysine content on HD-albumin. Competition experiments revealed that chlorolysine and carbamyllysine mediate binding of AOPP-albumin to SR-BI whereas binding properties of carboxmethyllysine did not differ significantly from native albumin.

## Conclusions

Oxidation and carbamylation of serum albumin generate relevant SR-BI antagonists in renal disease that may interfere with SR-BI-mediated reverse cholesterol transport. Displacement of HDL from its major receptor may result in decreased hepatic cholesterol uptake, depressed HDL metabolism and abnormal HDL composition and function. Dysfunctional reverse cholesterol transfer may contribute to the excessive cardiovascular mortality observed in patients suffering from renal disease.

